# The socioeconomic context of the use of *Euterpe precatoria* Mart. and *E. oleracea* Mart. in Bolivia and Peru

**DOI:** 10.1186/s13002-017-0160-0

**Published:** 2017-06-02

**Authors:** Narel Paniagua-Zambrana, Rainer W. Bussmann, Manuel J. Macía

**Affiliations:** 1 Herbario Nacional de Bolivia, Universidad Mayor de San Andrés, Casilla 10077 – Correo Central, La Paz, Bolivia; 2 William L. Brown Center, Missouri Botanical Garden, Box 299, St. Louis, MO 63166–0299 USA; 3 Departamento de Biología, Área de Botánica, Universidad Autónoma de Madrid, Calle Darwin 2, ES-28049 Madrid, Spain

**Keywords:** Agroforestry, Arecaceae, Asaí, Livelihoods, Non timber forest products, Palm-heart, Traditional knowledge

## Abstract

**Background:**

Traditional knowledge (TK) has enabled communities to adapt to changes in life conditions over centuries. However, this local context is being dramatically affected by recent changes through globalization and modernization of societies. In this paper we seek to identify socio-economic factors that are related to the knowledge and use of two palm species in mestizo and indigenous communities in the Amazonian of Peru and Bolivia. Both species are known in the region under the main vernacular name of *Asaí*, and are source of two highly commercialized resources: palm-hearts and fruits. *Euterpe precatoria* Mart. is native to the region whereas *E. oleracea* Mart. is being introduced for the use of both resources.

**Methods:**

We compare TK about the two *Euterpe* species in both countries in relation to 14 socioeconomic factors in seven use categories. We performed a Multivariate analysis of variance (MANOVA) to elucidate the influence of each socioeconomic factor on the overall palm knowledge or on individual use categories.

**Results:**

In the two countries, we found that mestizos, speaking only Spanish, and migrants in the same ecoregion, knew more uses in all use categories for *E. precatoria* than for *E. oleracea*, even in use categories such as Medicinal and veterinary and Construction, for which indigenous participants had more uses in case of other species. In Peru, the use of *E. precatoria* was higher among participants with greater wealth, which could be related to the commercial importance that both the fruits and the palm-hearts have had in the markets of the region. In contrast, in Bolivia, although some income generation from *Euterpe* sp. was observed, the use of *E. precatoria* was much more homogeneously distributed. The use of *E. oleracea* in Bolivia is recent, and although its most important uses are related to the consumption of fruits and palm-hearts (Human food), it is now being slowly used for Medicinal and Construction purposes, similar to *E. precatoria*.

**Conclusions:**

The use of each of the species forms part of divergent strategies in people’s livelihoods. We show that integration into a market economy does not necessarily erode TK, but can rather stimulate knowledge acquisition and transmission of knowledge, and helps to understand the role and potential of these products to contribute to the livelihoods of households.

## Background

Rural people worldwide depend on forest products and services for their daily income [1, 2]. The importance of these timber and non-timber products for subsistence and welfare is capital as have been documented in various tropical regions (e.g. [3–5]). Besides their importance in daily life, forest resources are also a source of income and livelihoods in times of scarcity and emergency [4, 6]. To increase the revenue from forest products, it has also been perceived as a strategy to improve the income of the poorest households in rural areas [7, 8]. However, market demand for forest products and development of agroforestry systems have been significant elements underlying social and environmental change in the Amazon, with strong implications for resource use strategies and livelihoods of rural populations [9].

Many studies have identified the increasing exposure to market economies as one of the factors that could lead to changes in traditional knowledge (TK in the following) that allows people in rural areas to make use of forest resources [10–12]. This is due to the integration in external markets which leads mostly to specialized extraction concentrated only on certain products, a homogenization in agricultural activities, and the replacement of local products with products from abroad, resulting in a higher socioeconomic heterogeneity and undermining the existence of traditional common knowledge [11–15]. Other researchers have found that the different activities, through which local people are linked to the market, were associated with the conservation of their knowledge [16]. The integration into the market through the sale of timber and non-timber forest products was associated with a greater understanding and use of forest resources [17]. Indeed, the use of resources for income generation also depends on other factors that influence the knowledge and use of resources linked to the household level such family history, availability of labor and capital, or past experiences, and factors linked to the personal level such as gender, age, ethnicity or level of education [12, 18–21]. Understanding how the use of forest resources relates to rural incomes is essential for designing policies to support livelihoods and sustainable development incentives in these regions [22, 23].

Using palms (Arecaceae) as an example, we identified socio-economic factors related to both the knowledge and use of species that are known to be important as a source of products of commercial interest. Many species of palms are locally used for subsistence, without including them in a system of direct income generation [24, 25]. The market for palm products has been very dynamic and difficult to predict. With current trade volumes, several wild species cannot meet demand in a sustainable manner [26]. Thus, there will most likely be an increasing pressure to switch from extraction to agroforestry systems production and plantations [9, 14, 25, 27, 28].

**Fig. 1:**
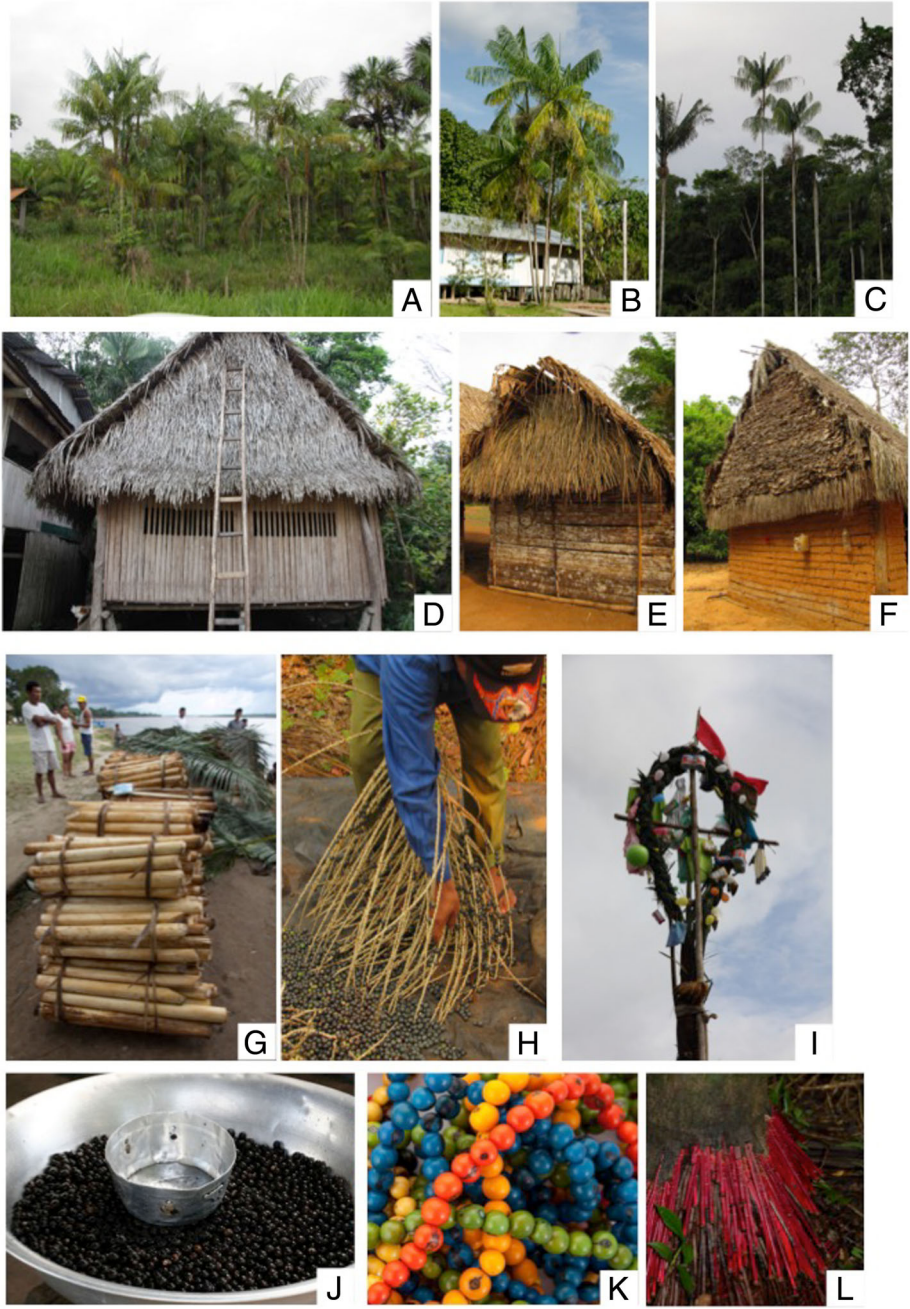
Presence and use of *E. precatoria* and *E. oleracea* in Amazonian communities in Peru and Bolivia. **a)** Plantation of *E. oleracea* in fallow field (Mestizos-Riberalta, Bolivia); **b**) *E. oleracea* planted as ornamental (Cocama, Peru); **c**) Individuals of *E. precatoria* in secondary forest close to the communities (Mestizos-Riberalta, Bolivia); **d**) House walls made from planks of the trunks of *E. precatoria* (Cocama, Peru); **e**) Walls made from split trunks, and roof made from leaves of *E. precatoria* (Chácobo, Bolivia); **f**) Edges of roof made from leaves of *E. precatoria* (Mestizos Riberalta, Bolivia); **g **) Palm hearts of *E. precatoria* cut for sale (Mestizos Iquitos, Peru); **h** and **j**) Harvest of ripe fruits of *E. precatoria* (Mestizos Riberalta, Bolivia); **i**) Umsha, a carnival ornament made from *E. precatoria* (Cocama, Peru); **k**) Dyed seeds of *E. precatoria* for jewelry production (Cocama, Peru); **l**) Root of *E. precatoria* used as medicine

Our research focused on the use of two species of neotropical palms of the genus *Euterpe* (*E. precatoria* Mart. and *E. oleracea* Mart.), which are known in the region under the main vernacular and commercial name *Asaí* [29, 30]*. E. precatoria* Mart. (Fig. 1a) is a solitary palm that occurs naturally below 2000 m elevation, on *terra firme* forests and along river banks, in periodically inundated areas growing from Belize in the North to Brazil and Bolivia in the South [29, 31]. The traditional use of *E. precatoria* has been reported frequently for house construction (e.g. posts, walls and for thatch), household utensils (e.g. fans, baskets, brooms), as well as medicine, but especially as food source (e.g. fruits and palm-hearts) [24] (Fig. 1d-l). In contrast *E. oleracea* Mart. (Fig. 1b-c) is a clonal species that grows in periodically inundated areas in Northern South America, in particular the Brazilian Amazon, the Orinoco basin, and in costal swamps of Colombia and Ecuador [29, 31]. Because of its economic importance as a source of palm-heart and fruits [25, 32], it has recently been introduced and used in different regions of Peru and Bolivia, outside its natural range ([32–34], Vincent Boss - Centro de Investigación y Promoción del Campesinado, personal communication). Until the early 1990s, the economic importance of these species was not recognized in Peru and Bolivia. After that, as result of industrial marketing, massive palm-heart exploitation started in natural populations of *E. precatoria*, until the early 2000s, when the market fell strongly [35–40]. Currently palm harvesting in both countries still exploits mostly the natural populations of *E. precatoria*, while *E. oleracea* is more and more cultivated and included in agroforestry systems [32, 33, 41]. Similarly, the market for fruits of both species has become more important during the last decade and asaí has changed from being a food for rural populations to an important product in large urban markets worldwide [25, 42, 43]. Its regional importance (as a raw material for the manufacture of beverages and ice cream) has promoted the commercialization of the fruits of *E. precatoria,* harvested in wild populations, and cultivation of *E. oleracea.* They constitute by now an important source of income ([25], Alvaro Torres - Madre Tierra S.L. de Amazonia/Instituto para el Hombre, Agricultura y Ecología, personal communication). The incorporation of *E. oleracea* arises as a sustainable alternative for the production of palm-hearts, because it is a multicaul species, as well as fruit harvest, because it has a high productivity per plant [33, 44, 45].

In this study, we comparatively evaluated the influence of 14 socioeconomic factors, at personal and family level, that have been associated with the palm TK in the region [20], on knowledge and uses of two species of *Euterpe* in indigenous and mestizo communities of Amazonian Peru and Bolivia.

We hypothesized that there would be differences in the use of *Euterpe* in different indigenous and mestizo communities, that *Euterpe* use in Peru would be more diverse than in Bolivia because the the native species (*E. precatoria*) is much more widespread, and the introduction of the commercial species (*E. oleracea*) occurred earlier, and that the socioeconomic factors affecting knowledge would reflect the differences in commercial importance of the species.

## Methods

### Data collection

**Fig. 2:**
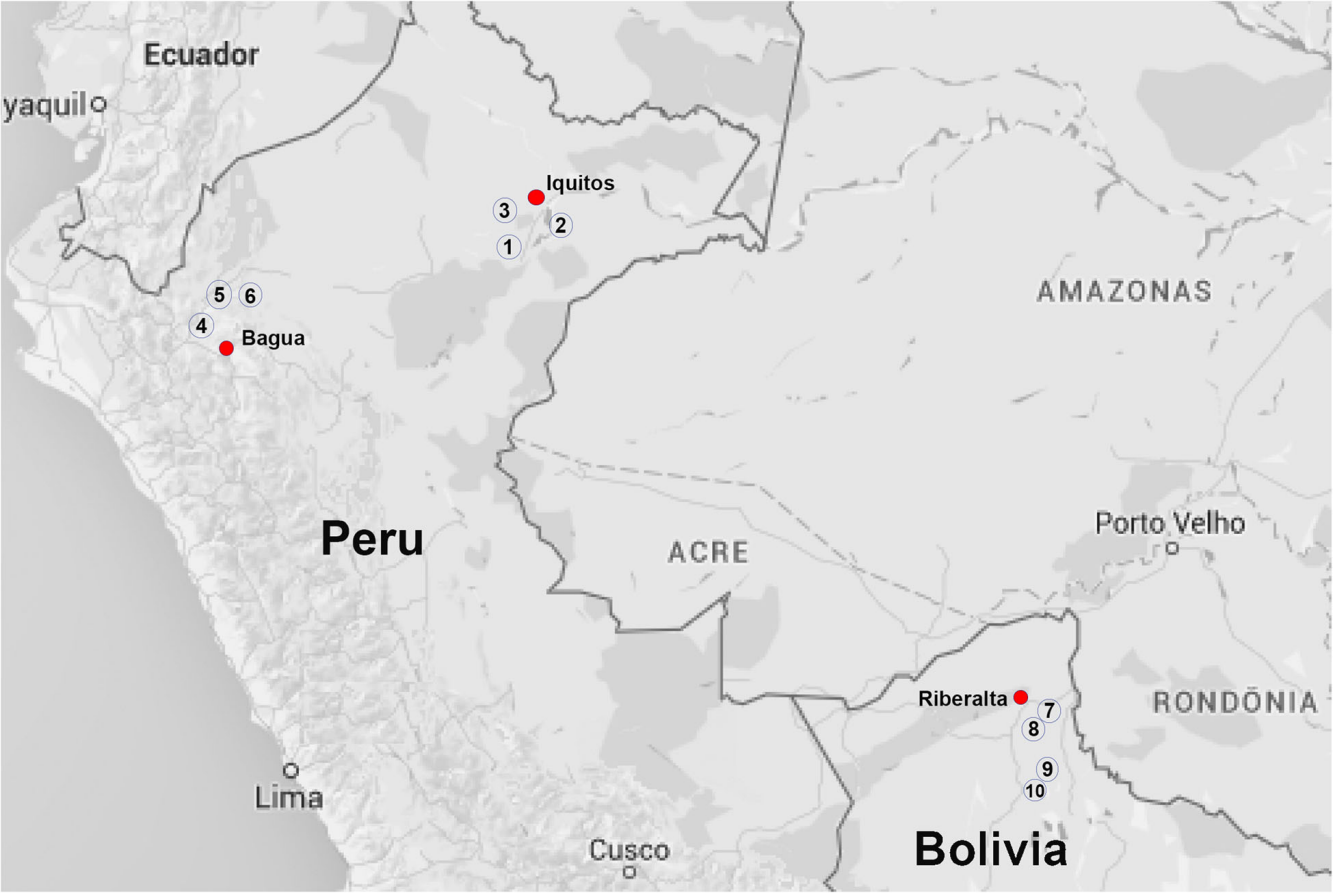
Map of the study areas in Peru and Bolivia showing the 10 communities where *Euterpe* use-data were recorded

Ethnobotanical data about the two species of *Euterpe* were gathered through semi-structured interviews using a standardized research protocol [46, 47]. Prior to starting interviews, we obtained collection and interview permits both from the respective national authorities, and each governing body of the indigenous and mestizo groups involved in the study. Before starting interviews prior informed consent was established with the communities in community meetings, and prior informed consent was also established with all individual informants. From March 2010 to December 2011, we interviewed 483 people in 10 communities inhabited by indigenous (*n* = 5) and mestizo (*n* = 5) groups in the Amazon of Peru and Bolivia (Fig. 2, Appendix 1).

Communities were selected to have a uniform ethnic composition and their divergent proximity to centers of commerce where products of both *E. precatoria* and *E. oleracea* were marketed. We divided informants into five age classes, starting at 18 years and using a range of 10 years for each age class (18–30, 31–40, 41–50, 51–60, and >60 years) to achieve an equal representation of all ages. Within the age classes, approximately 50% of people we interviewed were women and 50% were men (Table 1). Interviews were conducted in Spanish. In cases where an informant did not speak Spanish, the interviews were conducted with the help of local interpreters. We gathered socioeconomic information from all informants through structured interviews regarding seven socioeconomic variables concerning personal data: gender, age, ethnicity, education, languages spoken, migration status, time in residence, and seven factors concerning household data: size of family, tenure of farm animals, farm size, tools, transports, house size, house constructions materials (Table 2).

**Table 1:** Distribution of the 483 interviews conducted in 10 communities of Amazonian Peru and Bolivia by gender in five age groups. Additional data on the communities are shown in Appendix

Country	Ethnic group	# communities	Gender		Age (years)					# informants
			Men	Women	18-30	31-40	41-50	51-60	> 60	
Peru	Cocama	1	44	43	19	17	24	10	17	87
	Mestizo (Iquitos)	2	73	95	48	44	31	20	25	168
	Aguaruna	3	35	34	21	17	12	11	8	69
Bolivia	Mestizo (Riberalta)	2	39	40	10	18	19	14	18	79
	Chácobo	2	40	40	37	21	13	6	3	80
Total		10	231	252	135	117	99	61	71	483

**Table 2:** Description of the 14 socioeconomic variables gathered from 483 informants in 10 communities of Amazonian Peru and Bolivia

Independent variable	Variable type	Levels
Gender	Nominal	1) Men; 2) Women
Age	Continuous	Between 18 and 91 years
Ethnicity	Nominal	1) Indigenous; 2) Mestizo
Size of family (number of children)	Continuous	Between 0 and 18
Education (years)	Continuous	Between 0 and 16 years
Languages spoken	Nominal	1) Only native language; 2) Only Spanish; 3) Native language and Spanish
Migratory status	Ordinal	1) Non-migrant; 2) Migrant from other ethnic group in the same ecoregion; 3) Migrant from other ecoregion
Time in residence (years)	Continuous	Between 0.17 and 85 years
Farm animals	Ordinal	1) No animals; 2) Subsistence livestock; 3) Commercial livestock
Farm size (ha)	Continuous	Between 0 and 10 ha
Tools	Ordinal	1) Low cost (e.g. machetes, axes, bows and arrows, fishhooks, traditional agricultural tools); 2) Average cost (e.g. fishing-nets, carts, shotguns/rifles, plow, mechanical seed distributors); 3) High cost (e.g. fumigators, tractors, chainsaws, water pumps)
Transport	Ordinal	1) No transport; 2) No fuel consumption (e.g. canoe, bicycle); 3) Low fuel consumption (e.g. motorbike, small outboard motor); 4) High fuel consumption (e.g. truck, large outboard motor)
House size (m^2^)	Continuous	Between 10 and 272 m^2^
House construction materials	Ordinal	1) Local plant materials ≥50%; 2) Mixed material ≥50%; 3) Foreign commercial materials ≥50%

### Data analysis

We grouped the socioeconomic data obtained in the interviews into three types of variables: nominal (gender, ethnicity, languages spoken), ordinal (migration status, tenure of farm animals, tools, transports, house construction materials), and continuous (age, size of family, educations, time in residence, farm size, house size) (Table 2).

All palm-uses reported for both species of *Euterpe* in the interviews were classified in seven use categories following the Economic Botany Data Collection Standard [48] with some modifications proposed by Macía et al. [24]: Construction, Cultural, Environmental, Human food, Medicinal and veterinary, Utensils and tools, and Other uses (including indirect uses, especially the use of beetle larvae that develop in rotting trunks). The uses in categories were divided into subcategories to specifically analyze palm-uses. To determine the influence of socioeconomic factors on knowledge of both *Euterpe* species, we calculated the palm use-reports, representing the sum of all palm-uses reported by an informant for each of the two species of *Euterpe*. For this purpose, we use the definition of “palm-use” given by [24], which it is defines as the use associated to a use category and use subcategory for a specific plant part. To identify the socioeconomic factors associated to TK about both *Euterpe* species, we used the following ethnobotanical indicators: 1) Total palm use-reports; 2) The palm use-report in three use-categories in which both species had at least one reported use: a) Construction, b) Human food, and c) Medicinal and veterinary; and 3) The palm use-report in the two use subcategories of Human food: a) Beverage and b) Food, which was the only use-category that showed significant differences in relation to use knowledge.

To describe and compare TK about the both *Euterpe* species in relation to the 14 socioeconomic factors evaluated, we conducted (1) a descriptive analysis of the data set using a MANOVA (Multivariate analysis of variance) and its corresponding post hoc Tukey test for eight categorical variables (variables pertaining to less than 10 informants were not included in the analyses); and (2) Pearson correlations for the six continuous variables). All analyses were performed using JMP 11.0 (SAS Institute 2013).

## Results

### Uses of *Euterpe precatoria* and *E. oleracea* in Peruvian and Bolivian Amazon

A total of 70 palm-uses distributed in seven use categories and 30 subcategories were reported for the two species evaluated (Table 3). For *E. precatoria* we found 69 palm-uses (52 in Peru and 27 in Bolivia) and 2147 palm use-reports (63% and 37% in Peru and Bolivia respectively) in seven use categories in Peru and six in Bolivia. For *E. oleracea* we documented 17 palm-uses (16 in Peru and five in Bolivia) and 223 palm use-reports (64% and 36% in Peru and Bolivia respectively) in five use categories in Peru and three in Bolivia*.*

**Table 3:** Uses of *Euterpe precatoria* (A) and *E. oleracea* (B) gathered from 483 informants in 10 amazonian communities in Peru and Bolivia, distributed by ethnic groups and use categories. Use-reports are shown in parenthesis. (*) Local name for an ornament used during carnaval; (**) Axis used to form rubber bales (made from *Hymenaea courbaril*)

Palm use category	Palm use sub-category	Part plant used	Peru			Bolivia	
			Awajun	Cocama	Mestizo-Iquitos	Chácobo	Mestizo-Riberalta
A. Uses of *Euterpe precatoria*							
Construction	Houses	Stem	Roof frame (4) / Walls (4)	Floor frame (66) / Walls (87) / Flooring (6)	Roof frame (19) / Floor frame (2) / Walls (138) / Flooring (14) / Stake (6)	Walls (49)	Walls (71) / Flooring (6)
	Thatch	Entire leaf	Thatch (5)	Thatch (63)	Ridgepole (5) / Thatch (12)	Thatch (72)	Thatch (73) / Roof edge (4)
		Stem	-	-	Rods for thatch (6)	-	-
Environmental	Fences	Stem	-	-	Fences (1)	-	-
Human food	Beverages	Fruit	-	Juice (77)	Juice (136)	Juice (71)	Juice (77)
	Food	Fruit	Edible (39)	Edible (5)	Edible (47)	Edible (19)	Edible (4)
		Palm heart	Edible (45)	Edible (83)	Edible (163)	Edible (77)	Edible (79)
Medicinal and veterinary	Blood and cardiovascular system	Fruit	-	Anemia (4)	Anemia (1)	-	-
		Root	-	Anemia (1) / Blood pressure (2)	Anemia (3)	Anemia (4)	Anemia (72)
	Digestive system	Fruit	-	-	-	-	Diarrhea (6)
		Root	Hepatitis (2)	Liver pain (2)	Colic in babies (2) / Diarrhea (5) / Stomach pain (2) / Hepatitis (5) / Liver pain (2) / Gallbladder (2)	-	-
	Endocrine system	Root	-	Diabetes (3)	Diabetes (2)	-	-
	General ailments with unspecific symptoms	Fruit	-	-	Body weakness (2)	-	-
		Root	-	Fever (1)	Body pain (3) / Indisposition (2)	-	-
	Infections and infestations	Root	-	Malaria (25)	Malaria (33)	Amoebas (21)	Amoebas (7)
	Metabolic system and nutrition	Root	-	-	-	Malnutrition (2)	-
	Musculo-skeletal system	Root	-	Rheumatism and arthritis (1)	Hernia (2) / Rheumatism and arthritis (1)	-	-
	Poisonings	Stem	-	-	-	Snakebit antidote (7)	-
		Root	-	-	-	Snakebit antidote (2)	-
	Pregnancy, birth and puerperial	Root	Galactogogue (3)	Antiabortive (1)	Antiabortive (1) / Childbirth problems (5)	-	-
	Reproductive system and sex health	Root	-	-	Uterus infections (3) / Menstrual problems (5)	-	-
	Respiratory system	Stem	-	-	-	Pneumonia (1)	-
		Root	-	-	Bronchitis (1) / Pneumonia (14)	-	-
	Sensory system	Root	-	Ear ache (5)	-	-	-
	Urinary system	Root	-	Inflammation of kidneys (41)	Prostate (4) / Inflammation of kidneys (44)	-	-
	Other medicinal uses	Root	-	Hairloss (3)	Hairloss (2) / Stomach cancer (2)	-	-
	Not specified at all	Root	-	-	Unspecified (13)	-	-
Cultural uses	Clothes and accessories	Spear leaf	-	Hats (6)	-	-	Hats (8)
	Cosmetics	Fruit	-	-	-	-	Hair oil (2)
	Dyes	Fruit	-	-	Dyes (9)	-	-
		Root	-	-	Dyes (2)	-	-
		Spear leaf	-	-	Dyes (1)	-	-
	Personal adornment	Seed	-	Jewellery (9)	Jewellery (18)	-	Jewellery (1)
	Ritual	Entire plant	-	“Umsha” (2) (*)	-	-	-
Utensils and tools	Domestic and utensilis	Infrutescense	-	-	-	Broom (1)	-
		Leaf rachis	-	-	-	Broom (5)	-
		Spear leaf	-	Fan (10) / Basket (2) / Mats (1)	-	-	Fan (25) / Basket (2) / Mats (2)
		Stem	-	-	-	-	Mesons (3) / Shelf (6) / Ceiling (7)
	Hunting and fishing tools	Stem	-	-	Arc (2)	-	-
	Labor tools	Stem	-	-	-	-	“Tendal” (2)(**)
Other	Human food	Stem	Larvae edible (2)	-	-	-	-
	Medicinal	Stem	-	-	-	Larvae cough (7)	-
B. Uses of *Euterpe oleracea*							
Construction	Houses	Stem	-	-	Frame (6) / Walls (8) / Flooring (2)	-	Walls (1)
	Thatch	Entire leaf	Thatch (2)	-	Thatch (2)	-	Thatch (3)
Environmental	Ornamental	Entire plant	-	Ornamental (27)	-	-	-
Human food	Beverages	Fruit	-	-	Juice (8)	Juice (13)	Juice (41)
	Food	Fruit	Edible (17)	-	Edible (3)	-	-
		Palm heart	Edible (37)	-	Edible (10)	-	Edible (21)
Medicinal and veterinary	Blood and cardiovascular system	Root	-	-	-	-	Anemia (2)
	Digestive system	Root	-	-	Gallbladder (2)	-	-
	General ailments with unspecific symptoms	Fruit	-	-	Body weakness (2)	-	-
		Root	-	-	Indisposition (2)	-	-
	Pregnancy, birth and puerperial	Root	Galactogogue (3)	-	-	-	-
	Respiratory system	Root	-	-	Pneumonia (2)	-	-
	Urinary system	Root	-	-	Inflammation of kidneys (2)	-	-
	Not specified at all	Root	-	-	Unspecified (2)	-	-
Other uses	Human food	Stem	Larvae edible (5)	-	-	-	-

In Peru, for both species, the category Medicinal and veterinary was the one reporting the highest number of uses: 27 uses for *E. precatoria* and seven uses for *E. oleracea* (Table 3). In the case of *E. oleracea,* the use for Construction (four uses) and Human food (three uses), occupied the second and third position in order of importance. In the case of *E. precatoria,* the use for Construction (eight uses) and Cultural uses (six uses) were the second and third most important.

In Bolivia the use categories with the highest number of uses were Utensils and tools (nine uses), Medicinal and veterinary (seven uses), and Construction (four uses) for *E. precatoria* whereas for *E. oleracea,* Construction and Human food (both with two uses) were the most important (Table 3). In both countries, no use for *E. oleracea* in the use categories Utensils and tools and Cultural use was gathered. Most reported uses for both *E. precatoria* and *E. oleracea* were found in the mestizo communities in the region of Iquitos in Peru (43 and 13 uses respectively) and in the region of Riberalta in Bolivia (20 and five uses respectively) (Table 3). In both countries, all uses reported by an ethnic group for *E. oleracea* were also reported by the same ethnic group for *E. precatoria*. The only difference was Ornamental use, which was only reported by the Cocama in Peru.

### The significance of socio-economic variables in palm-use knowledge

Of the 14 socioeconomic factors assessed in both countries, three of them (gender, farm animals, and house construction materials) showed no significant difference, neither at total knowledge level, nor in the three use-categories (Table 4). In addition, the relationship with age and residence time were not significant in Peru, whereas the ownership of transport and the size of houses were not significant in Bolivia.

**Table 4:** Relationship between uses of *Euterpe precatoria* and *E. oleracea* (based on palm use-reports) and socioeconomic factors in the 10 Amazonian communities evaluated in Peru (A) and Bolivia (B)

	*Euterpe precatoria (*Mean ± SD)					*Euterpe oleracea (Mean ± SD)*			
	*n*	Total palm uses	Construction	Human food	Medicinal and veterinary	Total palm uses	Construction	Human food	Medicinal and veterinary
A. Peru									
Comparison of means (categorical variables)									
Gender									
Male	152	4.0 ± 1.9	1.4 ± 1.1	1.8 ± 0.7	0.8 ± 0.7	0.4 ± 1.1	0.1 ± 0.4	0.3 ± 0.6	0.1 ± 0.3
Female	172	3.9 ± 1.7	1.3 ± 1.0	1.8 ± 0.7	0.8 ± 0.7	0.3 ± 1.1	0.1 ± 0.4	0.2 ± 0.6	0.1 ± 0.3
Ethnicity									
Indigenous	118	3.2 ± 2.3 b	1.9 ± 1.3 b	1.5 ± 0.8 b	0.5 ± 0.7 b	0.5 ± 0.8 a	0.02 ± 0.1	0.5 ± 0.7 a	0.03 ± 0.1
Mestizo	206	4.4 ± 1.3 a	1.4 ± 0.8 a	2.0 ± 0.6 a	1.0 ± 0.6 a	0.3 ± 1.2 b	0.1 ± 0.4	0.1 ± 0.5 b	0.1 ± 0.02
Language spoken									
Only native language	6*	1.7 ± 1.8	0.3 ± 0.5	1.3 ± 0.8	-	0.5 ± 0.5	-	0.5 ± 0.5	-
Only Spanish	252	4.6 ± 1.4 a	1.6 ± 0.9 a	2.0 ± 0.5 a	1.0 ± 0.6 a	0.2 ± 1.1 b	0.1 ± 0.4	0.1 ± 0.4 b	0.1 ± 0.3
Native language and Spanish	66	1.7 ± 1.4 b	0.4 ± 0.8 b	1.2 ± 0.9 b	0.1 ± 0.4 b	0.8 ± 0.9 a	0.03 ± 0.2	0.7 ± 0.8 a	0.1 ± 0.2
Migration status									
Non-migrant	212	3.5 ± 1.9 b	1.1 ± 0.9 b	1.8 ± 0.8 b	0.7 ± 0.6 b	0.4 ± 0.9 b	0.04 ± 0.3	0.3 ± 0.6 a	0.03 ± 0.2
Migrant from other ethnic group in the same ecoregion	108	4.7 ± 1.3 a	1.8 ± 0.8 a	1.9 ± 0.5 a	0.9 ± 0.7 a	0.2 ± 1.2 a	0.1 ± 0.4	0.1 ± 0.4 b	0.1 ± 0.4
Migrant from other ecoregion	4*	6.3 ± 1.0	2.7 ± 0.5	2.3 ± 0.5	1.2 ± 0.5 b	1.7 ± 3.5	0.7 ± 1.5	0.7 ± 1.5	0.3 ± 0.5
Farm animal									
No animals	28	4.1 ± 0.3	1.3 ± 0.7	2.1 ± 0.5	0.7 ± 0.6	0.2 ± 0.6	0.03 ± 0.2	0.2 ± 1.1	-
Subsistence livestock	294	3.9 ± 0.1	1.4 ± 1.0	1.8 ± 0.7	0.8 ± 0.7	0.3 ± 1.0	0.1 ± 0.3	0.2 ± 0.6	0.1 ± 0.3
Commercial livestock	2*	5.0 ± 1.3	2.0 ± 0.0	2.0 ± 0.0	1.0 ± 0.0	5.0 ± 0.0	2.0 ± 0.0	2.0 ± 0.0	1.0 ± 0.0
Tools									
Low cost	58	2.5 ± 1.7 b	0.6 ± 0.7 c	1.6 ± 0.9	0.3 ± 0.5 b	0.4 ± 0.8	0.03 ± 0.2	0.4 ± 0.6	0.02 ± 0.1
Average cost	239	4.2 ± 1.7 a	1.4 ± 0.9 b	1.9 ± 0.6	0.9 ± 0.7 a	0.3 ± 1.2	0.1 ± 0.4	0.2 ± 0.6	0.1 ± 0.3
High cost	27	4.9 ± 1.5 a	2.1 ± 1.0 a	1.9 ± 0.6	0.8 ± 0.6 a	0.2 ± 0.9	0.1 ± 0.4	0.1 ± 0.4	0.04 ± 0.2
Transport									
No transport	88	2.3 ± 1.8 b	0.6 ± 0.8 b	1.4 ± 0.9 b	0.3 ± 0.5 b	0.5 ± 0.8	0.01 ± 0.1	0.5 ± 0.7 a	0.03 ± 0.2
Fuel									
No fuel consumption	108	4.6 ± 1.4 a	1.6 ± 0.9 a	2.1 ± 0.5 a	0.9 ± 0.6 a	0.2 ± 1.1	0.1 ± 0.5	0.1 ± 0.5 b	0.03 ± 0.2
Low fuel consumption	119	4.6 ± 1.4 a	1.7 ± 0.9 a	1.9 ± 0.5 a	0.9 ± 0.6 a	0.3 ± 1.2	0.1 ± 0.4	0.1 ± 0.5 b	0.1 ± 0.4
High fuel consumption	9*	4.7 ± 2.3	1.8 ± 1.4	1.8 ± 0.4	1.1 ± 1.2	0.7 ± 1.0	-	0.7 ± 1.0	-
House construction materials									
Local plant materials ≥50%	274	4.1 ± 1.7	1.4 ± 1.0	1.9 ± 0.6	0.8 ± 0.7	0.3 ± 0.9	0.1 ± 0.3	0.2 ± 0.6	0.02 ± 0.2
Mixed material ≥50%	2*	3.0 ± 0.0	1.0 ± 0.0	2.0 ± 0.0	-	3.0 ± 0.0	1.0 ± 0.00	2.0 ± 0.0	-
Foreign commercial materials ≥50%	48	3.4 ± 2.3	1.1 ± 1.0	1.6 ± 0.9	0.6 ± 0.7	0.6 ± 1.6	0.1 ± 0.4	0.4 ± 0.7	0.2 ± 0.6
Pearson correlation (continuous variables)									
Age	324	0.08	0.11	0.01	0.06	−0.09	−0.01	−0.1	0.02
Size of family (number of children)	324	0.1	0.11	0.03	0.09	−0.08	−0.08	−0.08	−0.04
Time in residence (years)	324	−0.02	−0.09	0.03	0.05	−0.1	−0.04	−0.1	0.03
Education	324	−0.11	−0.12	−0.09	−0.07	0.1	0.04	0.1	0.04
Farm size (ha)	324	0.15	0.17	−0.001	0.16	−0.02	0.08	−0.02	0.05
House size (m2)	324	0.24	0.16	0.23	0.3	−0.13	0.16	−0.14	0.12
B. Bolivia									
Comparison of means (categorical variables)									
Gender									
Male	79	4.7 ± 1.0	1.8 ± 0.5	2.1 ± 0.5	0.8 ± 0.5	0.4 ± 0.9	0.04 ± 0.3	0.4 ± 0.7	0.01 ± 0.1
Female	80	4.4 ± 1.2	1.7 ± 0.67	2.0 ± 0.1	0.7 ± 0.5	0.4 ± 0.8	0.01 ± 0.1	0.4 ± 0.7	0.01 ± 0.1
Ethnicity									
Indigenous	81	4.0 ± 1.3 b	1.5 ± 0.6 b	2.1 ± 0.6	0.5 ± 0.6 b	0.01 ± 0.1 b	-	0.01 ± 0.1 b	-
Mestizo	78	5.1 ± 0.6 a	1.9 ± 0.5 a	2.0 ± 0.3	1.1 ± 0.3 a	0.9 ± 1.0 a	0.1 ± 0.3	0.7 ± 0.8 a	0.03 ± 0.2
Language spoken									
Only native language	12	3.2 ± 1.9 c	1.4 ± 0.7 b	1.6 ± 0.9 b	0.2 ± 0.4 c	-	-	-	-
Only Spanish	73	5.0 ± 0.6 a	1.9 ± 0.5 a	2.1 ± 0.3 a	1.1 ± 0.3 a	0.9 ± 1.0 a	0.1 ± 0.3	0.8 ± 0.7 a	0.03 ± 0.2
Native language and Spanish	74	4.3 ± 1.2 b	1.6 ± 0.7 b	2.1 ± 0.5 a	0.6 ± 0.5 b	0.1 ± 0.3 b	-	0.1 ± 0.3 b	-
Migration status									
Non-migrant	84	4.1 ± 1.2 b	1.5 ± 0.6 b	2.1 ± 0.6	0.5 ± 0.6 b	0.01 ± 0.1 b	-	0.01 ± 0.1 b	-
Migrant from other ethnic group in the same ecoregion	71	5.0 ± 0.6 a	1.9 ± 0.4 a	2.0 ± 0.3	1.1 ± 0.3 a	0.9 ± 1.0 a	0.1 ± 0.3	0.8 ± 0.7 a	0.03 ± 0.2
Migrant from other ecoregion	4*	5.5 ± 0.6	2.5 ± 0.6	2.0 ± 0.0	1.0 ± 0.0	0.5 ± 1.0	-	0.5 ± 1.0	-
Farm animal									
No animals	7*	4.7 ± 0.5	1.8 ± 0.4	2.0 ± 0.0	0.8 ± 0.4	1.0 ± 0.9	-	1.0 ± 1.0	-
Subsistence livestock	145	4.5 ± 1.2	1.7 ± 0.6	2.0 ± 0.5	0.8 ± 0.6	0.4 ± 0.8	0.03 ± 0.02	0.3 ± 0.6	0.01 ± 0.1
Commercial livestock	7*	4.9 ± 0.7	1.7 ± 0.5	2.4 ± 0.5	0.7 ± 0.5	1.1 ± 0.9	-	1.1 ± 0.9	-
Tools									
Low cost	3*	5.0 ± 1.0	2.0 ± 0.0	2.3 ± 0.6	0.7 ± 1.5	0.3 ± 0.6	-	0.3 ± 0.6	-
Average cost	139	4.5 ± 1.1	1.7 ± 0.6	2.0 ± 0.5	0.7 ± 0.6	0.4 ± 0.7 b	0.01 ± 0.1	0.3 ± 0.6 b	0.01 ± 0.1
High cost	17	5.0 ± 0.9	1.9 ± 0.6	2.1 ± 0.6	1.0 ± 0.0	1.2 ± 1.2 a	0.1 ± 0.1	1.1 ± 1.0 a	-
Transport									
No transport	22	4.6 ± 0.6	1.7 ± 0.5	2.1 ± 0.2	0.9 ± 0.4	0.3 ± 0.63	-	0.3 ± 0.6	-
No fuel consumption	53	4.5 ± 1.0	1.7 ± 0.6	2.0 ± 0.5	0.8 ± 0.6	0.3 ± 0.5	-	0.2 ± 0.4	-
Low fuel consumption	80	4.5 ± 1.3	1.7 ± 0.7	2.0 ± 0.6	0.7 ± 0.5	0.0. ± 0.9	0.1 ± 0.3	0.4 ± 0.8	0.03 ± 0.01
High fuel consumption	4*	5.5 ± 0.6	2.0 ± 0.0	2.5 ± 0.6	1.0 ± 0.0	2.0 ± 0.01	-	2.2 ± 0.0	-
House construction materials									
Local plant materials ≥50%	146	4.5 ± 1.1	1.7 ± 0.6	2.1 ± 0.5	0.8 ± 0.6	0.4 ± 0.8	0.01 ± 0.1	0.4 ± 0.7	0.01 ± 0.1
Mixed material ≥50%	4*	5.5 ± 0.6	2.0 ± 0.0	2.5 ± 0.6	1.0 ± 0.0	1.5 ± 0.6	-	1.5 ± 0.6	-
Foreign commercial materials ≥50%	9*	4.7 ± 0.7	2.0 ± 0.0	1.7 ± 0.7	1.0 ± 0.0	0.7 ± 1.4	0.2 ± 0.7	0.4 ± 0.9	-
Pearson correlation (continuous variables)									
Age	159	0.44	0.45	0.07	0.33	0.2	0.05	0.2	0.01
Size of family (number of children)	159	0.42	0.28	0.15	0.36	0.34	0.08	0.34	0.07
Time in residence (years)	159	−0.09	−0.12	0.16	−0.15	−0.26	−0.07	−0.26	−0.05
Education	159	−0.09	−0.16	−0.03	0.07	0.02	0.14	0.02	0.08
Farm size (ha)	159	0.13	0.11	−0.16	0.15	0.19	0.18	0.19	0.16
House size (m2)	159	−0.03	−0.02	−0.05	0.07	0.05	0.15	0.04	0.13

Letters (a, b, c) indicate significantly different means based on a MANOVA analysis and its corresponding post hoc Tukey test (*p* < 0.05), with the levels indicated by different letters showing significant differences. (*) Levels with less than 10 replicas were not included in the analyses

In Peru, a total of six socioeconomic factors showed a different influence on the TK for both species: 1) ethnicity, with a higher knowledge of *E. precatoria* amongst mestizos and of *E. oleracea* among indigenous participants; 2) the language spoken, with higher knowledge of *E. precatoria* among people who only spoke Spanish and of *E. oleracea* amongst informants speaking both their native language and Spanish; 3) the possession of tools, with higher knowledge of *E. precatoria* among people owning average and high cost tools and without significant differences for *E. oleracea*; 4) ownership of means of transport, with higher knowledge about *E. precatoria* among participants that had transport means with no- or low- fuel consumption, but without differences in the case of *E. oleracea*; 5) farm size, with a significantly positive relationship for *E. precatoria* but non for *E. oleracea*; and 6) house-size, with a significantly positive relationship for *E. precatoria* and a negative relationship for *E. oleracea* (Table 4).

With regard to use-categories, both Construction and Medicinal and Veterinary use of *E. precatoria* showed a significant relationships with nine and seven factors respectively (Table 4). In both cases we found a higher knowledge among: 1) mestizos; 2) people who only spoke Spanish; 3) migrants from other ethnic groups within the same ecoregion; and among participants with 4) average and high cost tools; 5) transport with low and high fuel consumption; 6) larger farm size; and 7) larger house size. In addition, the use for Construction was higher among participants with larger families (more children) and a lower level of education. In contrast, knowledge of *E. oleracea* showed significant relationships only with the size of people’s houses, where higher knowledge corresponded to larger house size. Regarding the use for Human food, we found that both species showed the same significant differences for five factors, but with opposite patterns (Table 4). Higher knowledge about *E. precatoria* corresponded to: 1) mestizo people; 2) people who spoke only Spanish; 3) migrants from other ethnic group in the same ecoregion; 4) people who did own means of transport with fuel consumption; and 5) people with larger houses. The influence of these five factors on the two subcategories of Human food, showed that the knowledge about *E. precatoria* was clearly related with the use of its fruits to the production of beverages, while in case of *E. oleracea* the fruit and palm-heart were just used for food (Table 5).

**Table 5:** Relationship between two subcategories of Human food use of *Euterpe precatoria* and *E. oleracea* (based on palm use-reports) and socioeconomic factors in the 10 Amazonian communities evaluated in Peru and Bolivia

	*n*	PERU				*n*	BOLIVIA			
		*Euterpe precatoria*		*Euterpe oleracea*			*Euterpe precatoria*		*Euterpe oleracea*	
		Beverages	Food	Beverages	Food		Beverages	Food	Beverages	Food
Comparison of means (categorical variables)										
Gender										
Male	152	0.7 ± 0.5	1.2 ± 0.6	0.02 ± 0.1	0.2 ± 0.6	79	0.9 ± 0.2	1.1 ± 0.4	0.3 ± 0.5	0.1 ± 0.3
Female	172	0.7 ± 0.5	1.2 ± 0.6	0.03 ± 0.2	0.2 ± 0.5	80	0.9 ± 0.3	1.1 ± 0.4	0.3 ± 0.4	0.1 ± 0.3
Ethnicity										
Indigenous	118	0.4 ± 0.5 b	1.1 ± 0.7	-	0.5 ± 0.1 a	81	0.9 ± 0.3 b	1.2 ± 0.5	0.01 ± 0.1 b	-
Mestizo	206	0.8 ± 0.4 a	1.2 ± 0.5	0.04 ± 0.2	0.1 ± 0.03 b	78	0.9 ± 0.2 a	1.1 ± 0.2	0.5 ± 0.4 a	0.3 ± 0.4ª
Language spoken										
Only native language	6*	-	1.3 ± 0.8	-	0.5 ± 0.4	12	0.7 ± 0.5 b	0.8 ± 0.6 b	-	-
Only Spanish	252	0.8 ± 0.4 a	1.2 ± 0.4	0.03 ± 0.2	0.1 ± 0.3 b	73	0.9 ± 0.2 a	1.1 ± 0.2 b	0.5 ± 0.4 a	0.3 ± 0.4 a
Native language and Spanish	66	0.1 ± 0.2 b	1.2 ± 0.9	-	0.7 ± 0.8 a	74	0.9 ± 0.3 ab	1.2 ± 0.4 a	0.04 ± 0.2 b	0.03 ± 0.2 b
Migration status										
Non-migrant	212	0.6 ± 0.5 b	1.2 ± 0.6	0.01 ± 0.1	0.3 ± 0.6 a	84	0.9 ± 0.3 b	1.2 ± 0.5	0.01 ± 0.1 b	-
Migrant from other ethnic group in the same ecoregion	108	0.8 ± 0.4 a	1.1 ± 0.5	0.04 ± 0.2	0.1 ± 0.2 b	71	0.9 ± 0.1 a	1.0 ± 0.3	0.5 ± 0.4 a	0.3 ± 0.5
Migrant from other ecoregion	4*	1.0 ± 0.0	1.3 ± 0.5	0.3 ± 0.5	0.5 ± 1.0	4*	1.0 ± 0.01	1.0 ± 0.0	0.3 ± 0.5	0.3 ± 0.5
Farm animal										
No animals	28	0.7 ± 0.5	1.4 ± 0.5	-	0.2 ± 0.5	7*	1.0 ± 0.0	1.0 ± 0.0	0.4 ± 0.5	0.6 ± 0.5
Subsistence livestock	294	0.7 ± 0.5	1.2 ± 0.6	0.02 ± 0.1	0.2 ± 0.5	145	0.9 ± 0.02	1.9 ± 0.4	0.2 ± 0.4	0.1 ± 0.3
Commercial livestock	2*	1.0 ± 0.0	1.0 ± 0.0	1.0 ± 0.0	1.0 ± 0.0	7*	1.0 ± 0.0	1.4 ± 0.5	0.7 ± 0.5	0.4 ± 0.5
Tools										
Low cost	58	0.3 ± 0.5 b	1.3 ± 0.7	-	0.4 ± 0.6 a	3*	1.0 ± 0.0	1.3 ± 0.6	0.3 ± 0.6	-
Average cost	239	0.7 ± 0.5 a	1.2 ± 0.5	0.03 ± 0.2	0.2 ± 0.5 ab	139	0.9 ± 0.2	1.1 ± 0.4	0.2 ± 0.4	0.1 ± 0.3 b
High cost	27	0.8 ± 0.4 a	1.1 ± 0.5	0.04 ± 0.2	0.04 ± 0.2 b	17	0.9 ± 0.3	1.2 ± 0.4	0.5 ± 0.4 a	0.5 ± 0.5 a
Transport										
No transport	88	0.2 ± 0.4 b	1.2 ± 0.1	-	0.5 ± 0.7 a	22	1.0 ± 0.05	1.1 ± 0.2	0.2 ± 0.4	0.1 ± 0.3 ab
No fuel consumption	108	0.8 ± 0.4 a	1.2 ± 0.5	0.03 ± 0.2	0.1 ± 0.3 b	53	0.9 ± 0.03	1.0 ± 0.3	0.3 ± 0.5	0.02 ± 0.1 b
Low fuel consumption	119	0.8 ± 0.4 a	1.2 ± 0.1	0.04 ± 0.2	0.1 ± 0.3 b	80	0.9 ± 0.03	1.2 ± 0.4	0.3 ± 0.4	0.2 ± 0.4 a
High fuel consumption	9*	0.7 ± 0.5	1.1 ± 0.2	-	0.7 ± 1.0	4*	1.0 ± 0.1	1.5 ± 0.6	1.0 ± 0.0	1.0 ± 0.0
House construction materials										
Local plant materials ≥50%	274	0.7 ± 0.5	1.2 ± 0.5	0.02 ± 0.1	0.2 ± 0.5	146	0.9 ± 0.2	1.1 ± 0.4	0.3 ± 0.4	0.1 ± 0.3
Mixed material ≥50%	2*	-	2.0 ± 0.0	-	2.0 ± 0.0	4*	1.0 ± 0.0	1.5 ± 0.6	1.0 ± 0.0	0.5 ± 0.6
Foreign commercial materials ≥50%	48	0.5 ± 0.5	1.1 ± 0.7	0.04 ± 0.3	0.4 ± 0.7	9*	0.8 ± 0.4	0.9 ± 0.6	0.2 ± 0.4	0.2 ± 0.4
Pearson correlation (continuous variables)										
Age	324	0.02	0.1	−0.03	−0.1	159	0.23	−0.09	0.19	0.13
Size of family (number of children)	324	0	0.21	−0.1	−0.07	159	0.2	0.07	0.33	0.26
Time in residence (years)	324	−0.01	−0.12	0.01	−0.1	159	−0.02	0.21	−0.27	−0.12
Education	324	−0.07	0.07	−0.02	0.1	159	0	0	0	0.12
Farm size (ha)	324	0.05	0.2	0.07	−0.03	159	−0.02	−0.14	0.17	0.2
House size (m2)	324	0.33	0.16	0.11	−0.15	159	0.02	−0.09	0.02	0.19

Letters (a, b, c) indicate significantly different means based on a MANOVA analysis and its corresponding post hoc Tukey test (*p* < 0.05), with the levels indicated by different letters showing significant differences. (*) Levels with less than 10 replicas, not included in the analyses

In Bolivia, we found six factors with similar patterns for both species: 1) mestizos had a higher knowledge; 2) people who spoke only Spanish had a higher knowledge; 3) people belonging to other ethnic groups who had migrated in the same ecoregion knew more; 4) older participants had higher knowledge than younger; 5) people with larger families showed more knowledge; but 6) there was no significant relationship between knowledge and the level of education people held (Table 4). The three factors that showed a different influence over the total knowledge of both species were: 1) the time of residence, with a significantly negative relationship only in case of *E. oleracea*; 2) tool-ownership, with higher knowledge about *E. oleracea* linked to people with high-cost tools, but without significant differences for *E. precatoria*; and 3) Farm size, with a significantly positive relation only for *E. oleracea* (Table 4).

With regard to use-categories, both Construction and Medicinal and Veterinary use of *E. precatoria* showed significant relationships with six and five factors respectively (Table 4). In both cases, higher knowledge was found among: 1) mestizos, 2) people who only spoke Spanish, 3) migrants from other ethnic groups within the same ecoregion, 4) older participants; 5) people with larger families, and only in the case of Construction among people with lower education. In contrast, a high knowledge about *E. oleracea* was only related to the ownership of larger cultivated areas. The use of *E. precatoria* for Human food only showed significant differences with relation to two factors, both shared with *E. oleracea*: 1) language spoken, with more knowledge between people who only spoke Spanish; and 2) time of residence, with more knowledge in informants who actually lived more time in a community, in opposition to the findings in *E. oleracea*. In addition, *E. oleracea* showed significant differences with relation to: 1) ethnicity, with a higher knowledge among mestizos; 2) migratory state, with a higher knowledge held by members of other ethnic groups who had migrated within the same ecoregion; 3) age, with higher knowledge linked to increasing age; 4) family size, with more knowledge in people with larger families; 5) tool-ownership, with more knowledge in informants with high-cost tolls; and 6) farm size, with a more extensive knowledge in people with larger land farms (Table 4). The influence of these eight factors on the two subcategories included in Human food showed that the knowledge of *E. oleracea* was related to the use of its fruits for both the elaboration of beverages as well as the harvest of palm-hearts, in contrast to *E. precatoria* where the fruits were only used to produce beverages (Table 5).

## Discussion

In general, we found that the influence of the 14 socioeconomic factors evaluated on the TK of both *Euterpe* species showed more differences in Peru than in Bolivia. Our study indicates that the higher use of *E. precatoria* in the Amazonian areas of Peru and Bolivia occurred mainly among mestizos, even in the categories such as Medicinal use and Construction, which in many other studies have been documented mostly for the indigenous population [21, 49–53]. The ability of the mestizo population to experiment and learn has already been documented in other studies, and has been interpreted as an effect of the extensive experience that mestizos might have with external resources, which could motivate their interest to learn and know about, and experiment with the resources available in their immediate environment [52, 54–57]. This capacity associated with language (Spanish) as a mechanism of socialization and interchange [58], and their capacity of mobility between communities and regions (migrants in the same ecoregion), favored by the experience and familiarity of people with their environment, foster the acquisition of new knowledge that could be useful allowing them adapt to their new environment [51].

In this study we measured wealth primarily as agricultural and livestock assets, to reflect the different productive practices in which people engaged. Socio-economic compensation with regard to investments in introduced agricultural practices, animal husbandry, purchase of tools, capital and labor, are part of the households subsistence strategies, and therefore influence the decision about the removal and use of natural resources as a source of income, and enhance the interests of preserving TK [3, 17, 59]. Many studies from the tropics indicate that the poorest households depend, sometimes entirely, on the extraction of forest resources for their livelihoods, due to low capital requirements of such activities [60]. However, other studies have identified the opposite situation, in which only those households with sufficient capital for equipment, transportation, and labor can have economic benefits to market forest resources [4].

Our results reveal two patterns in relation to the influence of factors related to the wealth of participants on the knowledge of *E. precatoria*. In Peru, increased knowledge and use of the species was more common among the wealthiest people with greater purchasing power, even with regard to Medicinal and Construction use, although the forest resource could easily be replaced when people have access to external resources or local alternatives such as external construction material, and access to medicines and health centers [49, 50, 61, 62]. This pattern could be interpreted as a result of the attitude of people towards their environment, acquiring and preserving knowledge that potentially could be useful [63]. In this case, it might be related to the influence of the growing market for the products of *Euterpe* species [64]. However, as in the case of Human food use, including the use of the two commercialized resources (fruits and palm-hearts), this pattern could also be related to the accessibility (type and distance), and market characteristics (size and diversity of products) to which people have access [65]. Communities in Peru needed means to transport products long distances to markets, often using transport with high fuel consumption ([66], see Appendix 1), thus limiting the potential for revenue [1, 67]. This probably caused that knowledge about market use of *Euterpe* products mainly remained in the hands of people with higher purchase power [14]. Unlike in Peru, we did not find the influence of wealth on knowledge of *E. precatoria* in Bolivia, probably because livelihood strategies in this region are based on multiple commercialization of forest products, none exclusively extensive, including Asaí, and agriculture surplus [68]. In addition, the conditions of market access were less difficult (time and type of access, see Appendix 1) and markets were smaller (moving less volume). Thus, market access and marketing of products did not require a large capital investment. Generally places where people can sell palm-hearts and fruits of Asaí, are limited in Bolivia [Alvaro Torres - Madre Tierra S.L. de Amazonia / Instituto para el Hombre, Agricultura y Ecología, personal communication].

With regard to *E. oleracea*, our work indicates that this species is beginning to get incorporated into the body of TK of indigenous and mestizo communities in both Peru and Bolivia. Although uses of both species related to Construction and Medicine have been reported from both countries, the use for Human food, including commercial use (i.e. fruits and palm-hearts) were the most important. In Peru, the knowledge and use of the species by the Aguaruna was only related to the dietary intake of fruits and palm-hearts, similar to *E. precatoria*. It is noteworthy that he Cocama only knew ornamental uses. This might be due the fact that they live within a protected area (National Park Pacaya Samiria),limiting income development, and projects to introduce new species for commercial exploitation. The use-pattern for *E. oleracea* found in Bolivia seems to have a clear relation to factors like possession of expensive tools, and larger areas of cultivation, which was not evident in *E. precatoria*. This might be related to an interest of the specific informants, principally mestizos, in cultivating this species as a source of income ([16], Vincent Vos - Centro de Investigación y Promoción del Campesinado and Alvaro Torres - Madre Tierra S.L. de Amazonia/Instituto para el Hombre, Agricultura y Ecología, personal communication) and the type of land tenure in mestizo communities (parceling) allowing greater possibility of incorporating cultivated species into agroforestry systems [37].

Although other studies have highlighted how particular and localized effects of socioeconomic factors on traditional knowledge can be [20], our present work allows to highlight that these patterns vary even if only analyzing their influence at the level of one species. Therefore, great care has to be taken with generalizations about the importance of different species that are part of the body of TK.

## Conclusions

Our work shows that the influence of socioeconomic factors on the traditional knowledge and use of *E. precatoria* and *E. oleracea* is highly localized. The differences found in the influence of the factors evaluated in the communities in Peru and Bolivia show how highly variable and dynamic traditional knowledge can be. The importance of *E. precatoria* in Peru is more related to the commercial importance of its fruits and palm-hearts, in contrast to Bolivia, where, although the commercialization of both resources generates some income, this is still not as important as income generated by other resources or activities. The homogeneity we found in the whole region, and among all ethnic groups with regard to knowledge about *E. oleracea* and its used in linkage to the socioeconomic factors evaluated, reflects how recent this knowledge really is, and also shows that although some knowledge can be transmitted through the processes of general social interaction (coexistence and knowledge sharing living in the community), other parts are acquired through individual experimentation or interest. This underlines that in this very specific case differences between ethnic groups had no play in the way *Euterpe* sp. and their uses were introduced in different regions of Amazonia. Our work has shown that integration into a market economy does not necessarily erode TK, but can rather stimulate knowledge acquisition and transmission, and helps to understand the role and potential of these products to contribute to the livelihoods of households. However, the fact that usage patterns are highly localized indicates the need for carefully planned intervention strategies. This suggests that development efforts that aim to improve forest product incomes in rural livelihoods need to consider the diversification in livelihood strategies, the contribution of forest products in each of the livelihood strategies, and the sustainable livelihood assets that characterize a particular livelihood strategy.

## Appendix

**Table 6:** Characteristics of the 10 communities in the Amazon of Peru and Bolivia where 483 interviews about *Euterpe precatoria* and *E. oleracea* uses were gathered

N° Comunity	Community (Population)	Ethnic group	Accessibility^(a)^		Social services available^(b)^			Number of informants
			Nearest town (Population)	Type of access (Distance in hr.)	Electricity	Education	Healthcare	483
Peru								
1	San Martín (500)	Cocama	Iquitos (450,000)	Fluvial / Road (19)	3	2	3	87
2	El Chino (550)	Mestizo	Iquitos (450,000)	Fluvial (10)	1	1	2	79
3	Santa Ana (450)	Mestizo	Iquitos (450,000)	Fluvial (5)	2	2	1	89
4	Yamayakat (1000)	Aguaruna	Bagua (74,000)	Fluvial / Road (3)	3	1	3	36
5	Cusu Chico (150)	Aguaruna	Bagua (74,000)	Fluvial / Road (3.5)	3	1	3	13
6	Nueva Samaria (80)	Aguaruna	Bagua (74,000)	Fluvial / Road (3.25)	1	1	1	20
Bolivia								
7	Santa María (250)	Mestizo	Riberalta (90,000)	Loose surface road (1)	3	1	3	41
8	26 de Octubre (180)	Mestizo	Riberalta (90,000)	Road (1)	1	1	2	38
9	Alto Ivón (500)	Chácobo	Riberalta (90,000)	Road (4)	1	2	3	56
10	Motacuzal (30)	Chácobo	Riberalta (90,000)	Road (3.5)	1	1	2	24

^(a)^The information about the nearest town and the time to reach it were obtained from the interviews. The data represents the town and the time most frequently reported by the informants; ^(b)^Availability of electricity: (1) no electricity, (2) free electricity, (3) paid electricity. Access to education: (1) primary school, (2) secondary school. Access to healthcare: (1) without health post (only traditional medicine), (2) health post / community nurse, (3) health post / physician
